# Pediatric procedural sedation and analgesia

**DOI:** 10.4103/0974-2700.43189

**Published:** 2008

**Authors:** James R Meredith, Kelly P O'Keefe, Sagar Galwankar

**Affiliations:** 1 Department of Otolaryngology, University of South Florida, Tampa, Florida, USA; 2 Department of Medicine, Division of Emergency Medicine, University of South Florida, Tampa, Florida, USA; 3 Department of Global Health, University of South Florida, Tampa, Florida, USA

**Keywords:** Analgesia, pediatric, sedation

## Abstract

Procedural sedation and analgesia (PSA) is an evolving field in pediatric emergency medicine. As new drugs breach the boundaries of anesthesia in the Pediatric Emergency Department, parents, patients, and physicians are finding new and more satisfactory methods of sedation. Short acting, rapid onset agents with little or no lingering effects and improved safety profiles are replacing archaic regimens. This article discusses the warning signs and areas of a patient's medical history that are particularly pertinent to procedural sedation and the drugs used. The necessary equipment is detailed to provide the groundwork for implementing safe sedation in children. It is important for practitioners to familiarize themselves with a select few of the PSA drugs, rather than the entire list of sedatives. Those agents most relevant to PSA in the pediatric emergency department are presented.

## INTRODUCTION

The ability to provide safe and effective sedation and analgesia is an important skill for physicians involved in pediatric emergency care. Children are more prone to anxiety in the acute setting and benefit greatly from sedation and analgesia. Despite this, it is often underused due to misconceptions and unfamiliarity with the drugs or the procedure. There has been a movement to abandon the term “conscious sedation” due to its confusing and imprecise connotation.[1] Procedural sedation and analgesia, or PSA, has been proposed as a more appropriate term.[2]

Though restraining devices play a useful role in the pediatric emergency department (ED), there may be circumstances where a more elegant approach is waranted. Physicians have an large armamentarium of drugs with which they can calm the pediatric patient and ease the discomfort of unpleasant procedures. Physicians remain reluctant to administer PSA for many reasons; these include a fear of inducing deep sedation (and therefore hypoxia), time constraints, or a reluctance to overextend nursing personnel. A review of the literature shows that currently available agents have excellent safety profiles when administered in the correct doses, under close supervision and monitoring, and with the proper resuscitative measures at hand.[3–7] Krauss and Pena examined adverse events in 1,180 pediatric ED patients undergoing PSA and reported a 2.3% incidence of minor and transient incidents. In their series of patients, no serious complications occurred.[7] Even though sedating a patient may seem at times like an impedance to efficient medical care, it is beneficial to the patient and the family, as well as the physician.

*Procedural sedation* is the delivery of sedating or dissociative medications to produce a state of depressed consciousness, with or without opioid analgesics. PSA should allow the patient to maintain continuous and independent ventilation without a loss of protective reflexes. *Analgesia* is a loss of sensation to painful stimuli and is defined as having no effect on the sensorium. Most analgesics, however, tend to impair a patient's cognition as a side-effect. *Anxiolysis*, otherwise known as “anxiety alleviation”, tends to decrease a patient's apprehension without a decrease in the level of consciousness. The word *dissociative* refers to the encephalographic changes which demonstrate limbic and corticothalamic system disruption. This dissociative state results in analgesia, sedation, amnesia, and maintenance of muscle tone. *Sedation* encompasses a wide range of levels of consciousness, but it is less important to delineate the varying degrees of sedation than to recognize that sedation occurs along a continuum. [Table 1] outlines the varying levels of sedation.[8] Even during the course of a single PSA, a patient will fluctuate in and out of different levels of consciousness.

**Table 1:** Definitions of levels of sedation

Minimal sedation
Patient responds to verbal commands
Cognitive function may be impaired
Respiratory and cardiovascular systems unaffected
Moderate sedation and analgesia (f.k.a. “Conscious sedation”)
Patient responds to verbal commands, may not respond to light tactile stimulus
Cognitive function is impaired
Ventilation usually adequate, cardiovascular unaffected
Deep sedation and analgesia
Patient cannot be easily aroused without repeated or painful stimuli
Ability to maintain airway may be impaired
Spontaneous ventilation may be inadequate, cardiovascular function maintained
General anesthesia
Loss of consciousness, patient cannot be aroused even with painful stimuli
Adequate airway usually cannot maintained and ventilation is impaired
Cardiovascular function may be impaired

## INDICATIONS

Sedation has many uses in the pediatric ED setting. Severe pain, pain associated with procedures, and anxiety associated with diagnostic imaging can be effectively managed with sedation and analgesia. [Table 2] outlines common indications for PSA in the ED. Though clinical judgment should always prevail, PSA may be necessary in cases that may seem “minor” or relatively painless, but can still be quite traumatic for the pediatric patient. Sedation and analgesia should therefore be considered for most patients and offered as a choice to parents of patients undergoing a painful or unpleasant procedure. Satisfaction scores are higher for the patient, their families, and the physician in these cases. The desired depth of sedation is largely determined by the type of procedure being performed and the age of the child. Many procedures performed in the ED require deeper sedation. This includes procedures that are particularly painful or that require patients to remain motionless.

**Table 2:** Examples of common indication for PSA

Endoscopy
Wound dressing changes
Burn care
Orthopedic manipulation
Laceration repair
Suture removal
Lumbar puncture
Chest tube
Fecal impaction removal
Eye injuries
Central line placement
Forensic exams in cases of childhood sexual assault cases
Abscess incision and drainage

PSA: Procedural Sedation and Analgesia

## PATIENT PREPARATION

Appropriate patient selection is vital to successful PSA. Safety is, of course, the utmost concern. Careful investigation must be undertaken to assess the overall health status of each child. Because sedation in the emergency room is not appropriate for all patients, occasionally the procedure is better handled in the operating room under the care of an anesthesiologist. Severe systemic disease or life-threatening conditions are contraindications for PSA in the ED. [Table 3] outlines the physical-status classification of the American Society of Anesthesiologists used in sedation and analgesia for adults and pediatrics. Only patients that fall under the categories of Classes I and II are considered appropriate patients for PSA. Class III patients may be potential candidates after consultation with an anesthesiologist familiar with pediatric sedation.[9]

**Table 3:** American society of anesthesiologists physical status classification

Class	Description
I	A normal healthy patient
II	A patient with mild systemic disease
III	A patient with severe systemic disease
IV	A patient with severe systemic disease that is a constant life threat
V	A moribund patient, not expected to survive without the procedure

A thorough patient assessment is crucial before any procedure. Key aspects of the history prior to PSA are past medical history, chronic illnesses, allergies, current medications, drug use (including alcohol), last oral intake, anesthetic history, events leading up to this presentation, and volume status [Table 4].

**Table 4:** Key components of history to ask a patient prior to PSA

Past medical history:
Major illnesses—Asthma (*histamine release with morphine*), psychiatric disorders (*emergence reactions with ketamine*), cardiac disease (*Hemodynamic properties of certain sedatives*), hepatic or renal impairment (*increased toxicity decreased clearance*), or porphyria (*etomidate*).
Allergies:
Opitaes, benzodiazepines, barbiturates, local anesthetics, or others. *An allergy to one class of opiates does note generally confer cross-allergy to other classes of opiates. For example, codeine and morphine are natural opioids, whereas fentanyl is synthetic*.
Current medications:
Cardiovascular medications, CNS depressants, *May alter vital signs, volume status, or resuscitative measures. Be careful with chronic benzodiazepine and opiate users—administration of reversal agents may induce withdrawal or seizures*.
Drug use:
Narcotics (including heroin), benzodiazepines, barbiturates, cocaine, and alchol. *May make the experienced patient more tolerant of standarad doses of sedatives and analgesics compared to the naïve patient*.
Last oral intake:
For non-emergent cases, some guidelines recomment >6 hours for solid food and > 2 hours for clear liquid.[10] However, fasting versus non-fasting has not shown to change adverse events.[11]
Volume status:
Vomiting, diarrhea, fluid restriction, urine output, making tears. *(Dehydration may potentiate hypotensive side-effects of some agents)*.

PSA: Procedural Sedation and Analgesia

Preparing the patient for the procedure and PSA begins with a full detailed explanation of both the procedure and sedation, including risks, benefits, and potential side effects. Patients old enough to drive also need to be forewarned that they will be unable to drive after sedation and need to arrange for transportation. An informed consent for the procedure *and* the PSA needs to be signed by the legally responsible person. A thorough explanation of the events that will be occurring and taking the time to answer the parent's questions, is not only required ethically and legally, but can also alleviate concerns and have a calming effect.

The intravenous route of administration is very popular among pediatric ED physicians. Though it has not been shown to be safer, intravenous (IV) access has potential safety features over other routes, as sedating and analgesic medications can be administered slowly and titrated to effect. IV access also allows for rapid delivery of resuscitative and reversal agents should an adverse reaction occur. Hypotension can be prevented or reversed in some cases with intravenous hydration. However, a recent review of the literature reveals that last oral intake status has no effect on complications, including aspiration.[9,11] Oral sedation is also useful and avoids the anxiety associated with needle sticks, but patients undergoing painful procedures or requiring a deeper state of sedation are best handled by other routes of administration. Medications and available routes of administration are discussed in detail later in the article.

The criteria for patient discharge following PSA are straightforward, but are crucial to ensure patient safety. Patients should have stable vital signs, including no evidence of hypotension or hypoxia. Patients should return to a level of orientation and consciousness similar to their pre-procedural state. Patients should be offered fluids before discharge to assess whether they can tolerate drinking without emesis. The times of greatest concern for adverse events are five to 10 minutes following the last administration of sedation *and* after the painful stimuli from the procedure have been removed. Newman *et al.* found that in a series of 1,367 patients, that if there were no adverse events during the PSA, then patients could safely be discharged after 30 minutes of receiving the last dose of medication.[12]

## EQUIPMENT

Procedural sedation can only take place with the proper provisions for monitoring. Those serving pediatric patients need a wide range of supplies to fit the varying sizes of patients [Table 5]. Vascular access may be preferred for administering PSA, but no matter which route is used, supplies must be available to secure venous access in case of an emergency or when resuscitation is needed. Hypoxia is one of the most common side effects of PSA and can be effectively prevented and treated with oxygen, either by face-mask or nasal canula. Hypoventilation can also be remedied with the use of positive airway pressure with a bag-valve mask when a patient experiences respiratory depression. Age-appropriate endotracheal tubes, laryngoscopes, suctioning devices, and bag-valve masks should be available should more emergent airway management be required.[3,8,9,12–14]

**Table 5:** Equipment for procedural sedation and analgesia

High flow oxygen source and delivery device
Suction and large bor catheters
Vascular access materials
Airway management supplies: endotracheal tubes, bag valve masks, and laryngoscopes
Pulse oximetry, blood pressure device, electrocardiography*, capnography*
Resuscitation drugs, including intravenous fluids
Reversal agents, including flumazenil and naloxone

* May be Optional Devices

Patient monitoring is crucial in PSA—vital signs, including blood pressure, pulse, and respirations, should be recorded at regular intervals. Continuous pulse oximetry can serve as an excellent patient monitor for adequate oxygenation. For more optimal patient monitoring, electrocardiography and capnography are used during sedation. Capnography detects increasing levels of carbon dioxide before desaturation occurs and can detect early inadequate ventilation.[13] It is purported that capnography raises the standard of care, but studies have failed to show any significant decrease in preventing adverse events.[15] During PSA, the “code cart” should be easily accessible. The cart should be well stocked with all the needed materials, including resuscitation drugs. Reversal agents, such as flumazenil and naloxone, should be available whenever benzodiazepines and opiates are administered.

Of course, PSA is not possible without adequately trained and experienced personnel. PSA should only be done when nursing staff is undistracted and completely devoted to monitoring the patient. During the course of the PSA, the nursing staff should record the vital signs every five minutes for 30 minutes after the last dose. At the conclusion of the procedure, the patient must be closely monitored until there is a return to a full level of consciousness. The patient should not be left alone during this time for any reason, even if they seem awake.

A tool that may be of some interest for PSA is the potential use of the Bispectral Index (BIS) for evaluating the level of sedation. BIS has been proposed as an aid for physicians administering PSA in the ED. The BIS is useful to anesthesiologists for general anesthesia in the operating room as an objective measure of sedation. The BIS quantifies the depth of anesthesia using a processed electroencephalogram reading and converting the signal into a score on a scale of zero to 100, with zero being coma and 100 being awake. The Ramsay Sedation Score (RSS), which is considered the gold-standard for clinical assessment of sedation, is a subjective rating system used to document and assess the level of sedation on a scale of one to six, with one being agitated and six being unresponsive. Bell *et al.* found the optimal BIS for procedural sedation to be 80-85, and they corresponded a RSS grade of three (responsive only to verbal stimulus) and four (brisk response to glabellar tap or loud auditory stimulus) to a BIS of 87.2 and 80.9, respectively. They, like many authors, admit that further investigation is warranted to evaluate the usefulness of BIS in ED sedation.[16] Gill *et al.* compared the BIS to the RSS. They found that BIS was able to reliably tell the difference between general anesthesia and mild sedation. However, it had low discriminating power in the mild-to-moderate and moderate-to-deep levels of sedation.[17] At this point it seems that BIS may serve as an adjuvant to subjective assessments, but may not be able to decipher varying levels of sedation with any great certainty.[18] No studies to date have shown that BIS effectively alters outcome of PSA in the pediatric ED.

## PHARMACOLOGY OF PSA [Tables 6 and 7]

**Table 6:** Drugs used in procedural sedation and analgesia

Analgesics Class	Analgesics Name	Dose/Route	Peak	Duration
*Opiates*	Morphine	0.1-0.2 mg/kg IV	15 min	2-4 hours
	Fentanyl	1-2 mcg/kg IV	10 min	30-60 min
Sedatives				
*Benzodiazepines*	Midazolam	0.02-0.1 mg/kg IV	5 min	1-2 hours
		0.2-0.5 mg/kg IN	20-30 min	60-90 min
		0.5-0.75 mg/kg PO	20-30 min	60-90 min
*Barbiturates*	Pentobarbital	2-5 mg/kg PR(max 150 mg)	15-60 min	1-3 hours
		1-3 mg/kg IV (max 150 mg)	1 min	15 min
*Hypnontics*	Propofol	1 mg/kg IV, then 0.5 mg/kg IV over 30-60 min	6-7 min	5-10 min
	Etomidate	0.1-0.2 mg/kg IV	30 sec	5-10 min
Dissociative				
	Ketamin	1-2 mg/kg IV	5 min	30-60 min
		3-5 mg/kg IM	10 min	1-2 hours
*Adjuvants for ketamine*	Atropine	1 mg/kg IM/IV, min 0.1 mg (max 0.5 mg)	-	-
	Midazolamk	0.05mg/kg IV	-	-
*Inhaled*	Nitrous Oxide	30-50% N_2_O, mixed with O_2_	3-5 min	5-10 min
Reversal agents				
*Opioid reversal*	Naloxone	*<5yo or <2olbs:0.1 mg/kg* IV/IM/SC/ET q2-3 min	1-2 hours	-
		>5yo or >20 lbs: 2 mg IV/IM/SC/ET q2-3 min	1-2 hours	-
*Benzodiazepine reversal*	Flumazenil	0.02 mg/kg IV q 1min (max 1 mg)	-	-

**Table 7:** Commonly used combinations of drugs and their indications

Midazolam and Morphine (IV)
*Complex laceration repair*
*Incision and drainage with packing*
Ketamine and Midazolame (IV)
*Reduction of orthopaedic injuries*
*Lumbar puncture*
Ketamine (IM)
*Complex suture removal*
*Forensic exams*
Pentobarbital (PR)
CT scans, MRI, etc.

PSA is a blossoming field in Emergency Medicine. Emergency Medicine physicians caring for pediatric patients must have a sound understanding of sedation principles and safety issues surrounding the process and agents used. There is a mounting body of evidence on the topic of pediatric sedation. The objective of this paper was to screen the literature for up-to-date developments in PSA and present sound advice with a proven track record in the literature and clinical practice.

Depending on the procedure, PSA can involve monotherapy or combination-therapy. Each regimen and administration of PSA must be carefully tailored for each patient. Patient idiosyncrasies, including rate of metabolism, weight distribution, drug experience, etc., can have a profound impact on PSA. The drugs commonly used in PSA today have an excellent safety profile, however, their use must always be exercised with great caution. Each of the intravenous drugs must be titrated to effect. Overzealous administration may result in deep sedation or general anesthesia with depression of ventilation and hypoxia. When administered, the drugs should be given as an appropriate initial dose with subsequent doses until titrated to effect with 3-5 minutes between each bolus for the drug to take full effect. Patient responsiveness and vital signs should be assessed during PSA induction and throughout the course of the sedation.

### Local anesthetics

Although a thorough explanation of local anesthetic drugs and techniques is beyond the scope of this article, a quick note about their usefulness with PSA is warranted. Whenever possible, local anesthetics should be implemented along with PSA. The benefits of local infiltration are numerous, and can be a powerful adjuvant. Patients under sedation usually will still respond to painful stimuli. A local anesthetic can make the patient completely insentient to the pain. It can also allow for the use of less systemic analgesia, which may be safer. If a long-acting anesthetic, like bupivicaine, is used it can afford the patient prolonged pain relief after the procedure. Topical anesthetics may also be helpful. Formerly, a solution of tetracaine, adrenaline, and cocaine (TAC) was used. The adverse effects of cocaine toxicity make TAC an unfavorable choice, especially in areas of mucosal involvement. A mixture of equal parts lidocaine 4%, epinephrine 0.1%, and tetracaine 0.5% (LET) is highly effective. It is also safer than TAC and does not contain a controlled substance. LET and TAC should not used on end-arterial regions (ears, fingers, penis, etc.) due to its vasoconstriction. The LET solution should be soaked in a cotton ball, applied to affected area, and secured with adhesive tape for at least 20 minutes.[19]

### Opioids

Opioids have a significant effect on pain and possess sedative properties making them a standard of PSA. *Opium*, derived from the Greek word for 'juice', is a mixture of 20 different alkaloids from the opium poppy, *Papaver somniferum*. *Opioid* refers to all natural or synthetic exogenous ligands, which act as agonists on opioid receptors. Opioids mimic the activity of the endogenous ligands, *endorphins*, which is derived from the words “endogenous” and “morphine”. The analgesic effects result from suppression of spontaneous and evoked responses of neurons within the central nervous system along pain pathways.[20]

Morphine is a naturally occurring opioid and is considered the prototypical opioid agonist. Morphine is a good choice for PSA due to its desirable effects of being able to produce analgesia, euphoria, and sedation. The analgesic properties of morphine are most notable when given prior to a painful insult, which is particularly pragmatic in PSA. The long duration of morphine may be helpful if pain is anticipated after the procedure, however, it may also prolong somnolence. Side effects associated with morphine include nausea, feeling of warmth, heaviness of extremities, dry mouth, hypotension, and pruritus, especially of the face and nose. Morphine is conjugated with glucuronic acid primarily in the liver to make morphine 3-glucuronide and morphine 6-glucuronide. Glucuronidation allows for renal excretion of the metabolites. However, morphine 6-glucuronide is an active metabolite and can be the cause increased levels and subsequent ventilatory depression in patients with renal impairment. Exaggerated effects can also be seen in patients taking monoamine oxidase inhibitors, which disrupt the glucuronidation of morphine. Morphine causes hypotension by reducing sympathetic nervous system input resulting in peripheral venous dilatation, which decreases venous return. Morphine also stimulates the vagal nucleus in the medulla and can cause bradycardia. The most profound effect on hemodynamics may come from the release of histamine, which results in vasodilation. These effects may be blunted with administration of fluids in patients who may be slightly hypovolemic. Hypoxia is the side effect of most concern. Like all opioids, morphine causes a dose-dependent depression on the ventilatory centers in the brain stem. The hypoxia is a result of morphine to reduce responsiveness to increasing levels of carbon dioxide and interfering with breathing rhythm in the pontine and medullary ventilatory centers.[5,20,21]

Fentanyl is a synthetic opioid agonist with a rapid onset and short duration of action, making it an excellent choice for PSA. Fentanyl is about 100 times more potent than morphine, but has a wide therapeutic window. The other desirable attributes include the lack of direct of myocardial depressant effects and absence of histamine release. Like morphine, the major concern with fentanyl is the ventilatory depression. Caution must be exercised when administering fentanyl because of the association of chest wall rigidity following rapid, large boluses (>15 mcg/kg). Treatment of the rigid chest wall phenomena is pharmacological paralysis and mechanical ventilation. Fentanyl should be administered in 0.5 mcg/kg increments until adequate level of sedation is achieved. Fentanyl causes profound facial prutitus and is therefore cautioned for procedures involving the face.[20,22,23]

Meperidine (Demerol^®^) has grown out of favor in past years. It has a shorter half-life than morphine, but fentanyl is preferred as a short-acting opioid. Meperidine has less predictable sedative properties compared to morphine and fentanyl. The metabolites of meperidine are toxic to the central nervous system at high doses and in patients with renal impairment. Side effects of meperidine include a greater histamine release than seen with morphine, hypotension, and seizures. The CNS excitation associated with meperidine is not reversed by naloxone. Fatal reactions have also occurred in patients taking monoamine oxidase (MAO) inhibitors. Meperidine is not recommended for PSA in the ED.[20,21,23]

### Barbiturates

The class of barbiturates is becoming less popular in PSA as new sedatives and hypnotics make their way into the ED. Barbiturates cause sedation by depressing the reticular activating system. Barbiturates affect the rate of dissociation of the neurotransmitter gamma-aminobutyric acid (GABA) from inhibitory neurons mediated by the receptor. This keeps the ion channel open and allows for an influx of chloride ions, which hyperpolarizes the neuron and inhibits the postsynaptic neurons. The depression of transmission within the sympathetic nervous system ganglia may contribute to a decrease in blood pressure. The short duration of barbiturates is attributable to its rapid redistribution from the brain to inactive tissues, not necessarily from enzymatic metabolism. Barbiturates are cerebral vascular vasoconstrictors, which decrease blood flow to the brain and lowers increased intracranial pressure. Barbiturates have no intrinsic analgesic activity. Long acting barbiturates may be less preferable than benzodiazepines for PSA as there is no reversal agent for barbiturates.[13,19,20]

Pentobarbital is an ultra-short acting barbiturate which has become very useful for sedation prior to diagnostic imaging procedures in children. It can be given intravenously or rectally, but the rectal route has proven to be safe and well tolerated and is therefore preferred for this purpose. Continuous monitoring is still needed, however, especially if the patient leaves the ED for the procedure. Pentobarbital does not lower the seizure threshold as does methohexital. Adverse reactions include those similar to all CNS depressants, such as hypoxia due to a dose-dependant depression of the ventilatory centers in the medulla and pons. A paradoxical excitatory phenomenon has also been associated with barbiturate administration. The cardiovascular effects include a decrease in blood pressure with a compensatory increase in heart rate.[24,25]

### Benzodiazepines

Benzodiazepines are preferred agents because of their desirable profiles. Benzodiazepines have no analgesic activity but they are anxiolytic, amnesic, hypnotic, and act as a skeletal muscle relaxant. The anterograde amnesia is highly desirable and impairs the patient's ability to acquire and encode new information, such as that of a traumatic procedure. Benzodiazepines are also advantageous because they can be reversed with an antagonist, flumazenil. Flumazenil is a specific benzodiazepine antagonist and can reverse the CNS effects in about two minutes. Benzodiazepines modulate the GABA receptors via the alpha subunit and increase the frequency of the ion channel opening. Benzodiazepines have no appreciable effect on intracranial pressure, so are not contraindicated with intracranial pathology. Rapid, large boluses, especially in the presence of opioids can cause transient apnea. There is a decrease in blood pressure and an increase in heart rate associated with benzodiazepines, but they do not affect cardiac output.[20,26,27]

Midazolam has become the benzodiazepine of choice in PSA. It has a rapid onset of action and short duration of action compared to diazepam and does not have as many side effects. The anterograde amnesia can be a desired side effect, but care must be taken when discharging patients. In older pediatric patients, discharge instructions may not be remembered when they get home; be sure to write out specific instructions. Midazolam is approved for many routes, including oral and nasal and is most useful for anxiolysis and mild sedation. However, intranasal administration may be unpleasant and difficult to tolerate by the patient. Midazolam has a bitter taste and can be combined with noncitrus juice to make it more palatable. In alcohol users, larger doses of midazolam may be required to achieve the desired effect. Diazepam is not used for many reasons including delayed onset, long half-life, pain upon injection, and phlebitis.[5,19,20,26–30]

### Dissociative sedation

Ketamine is a phencyclidine (PCP) derivative and is classified as a *dissociative anesthetic* due to a disruption of cerebral association pathways. Ketamine is ideal because it demonstrates analgesic, amnesic, and sedative properties without a loss of protective reflexes. Patients given ketamine often look as if they are in a cataleptic state—their eyes remain open and there is a slow nystagmic gaze. They cannot communicate, but they may appear wakeful and make non-purposeful movements not associated with the procedure. Ketamine does increase cerebral blood flow and is therefore contraindicated in patients with increased intracranial pressure. Ketamine also induces salivary secretions, which can induce cough and laryngospasm. These effects can be attenuated with adjunctive use of an antisialagogue, such as atropine. The atropine is given 0.01 mg/kg, 0.1 mg minimum and 0.5 mg maximum, and can be given in the same syringe as the ketamine. Ketamine acts similarly to cocaine in that it increases sympathetic nervous system stimulation by inhibiting reuptake of catecholamines, which results in increased blood pressure, heart rate, and cardiac output. It does not usually, however, produce any appreciable depression of ventilation, but if given as a rapid bolus or concomitantly with opioids, apnea can occur. An important adverse reaction associated with ketamine is the emergence phenomenon, which can manifest as disturbing dreams or frightening hallucinations. It is most common in females, age greater than 16 years old, doses greater than 2 mg/kg IV, history of psychiatric disorders, and persons who normally dream. Midazolam given 5 minutes before the ketamine has been reported to prevent the emergence phenomena. However, several studies have reported no benefit of premedication with midazolam. If giving ketamine intramuscularly (IM), the ketamine, atropine, and midazolam can all be mixed in one syringe. Ketamine is contraindicated with increased intracranial or intraocular pressure, pregnancy, hyperthyroidism, or uncontrolled hypertension. As with all agents in PSA, ketamine must be slowly administered because rapid boluses have been associated with malignant arrhythmias. Ketamine given IV has not been shown to be safer than IM administration.[19,20,23,27,28,31–34]

### Hypnotics

Etomidate is a very rapidly acting sedative, causing unconsciousness within one arm-to-brain circulation time, and also has the benefit of a fast offset, resulting in rapid awakening. To be used for long procedures, etomidate should be administered as a continuous infusion. Etomidate, like barbiturates and benzodiazepines, has no analgesic activity. Nausea and vomiting are common side effects of after sedation with etomidate. In about one-third of patients, myoclonus occurs, but is benign and not considered hazardous. It is also associated with seizures in patients with epilepsy. Cardiovascular stability makes etomidate an attractive choice for sedation especially when a patient's cardiac status is in question. Etomidate lowers intracranial pressure by decreasing cerebral blood flow. It is not associated with histamine release. Etomidate has minimal effects on ventilation though these may be intensified with co-administered opioids or rapid infusion. There can be marked pain on intravenous injection thought to be due to the irritating effect of the solvent, propylene glycol.[35] The adrenocortical suppression often associated with long-term use probably has little impact on PSA.[23,36,37]

One of the hot-topics in the pediatric emergency medicine literature concerns the use of propofol in the ED. There has been some resistance to its use as an acceptable agent for PSA in the ED from anesthesiologists and emergency physicians.[38,39] Though most hospitals have sanctions against its use outside of the operating room, some institutions are evaluating its potential benefits. Bassett *et al.* evaluated propofol in pediatric sedation in the ED and found it was efficacious and safe.[40] Due to its rapid onset and offset, it was well-suited for short procedures in the ED. Patients receiving it required assisted ventilation at a rate of 0.8% compared to 0.3% for the fentanyl-midazolam group. The authors also point out that the most serious complication of PSA is aspiration, not respiratory depression, and that a "wait-out" period is possible with the use of propofol. The antiemetic properties of propofol also protects against the possible risk of aspiration. There is a transient cardiopulmonary depression that occurs with propofol administration, which requires vigilant monitoring by staff with the necessary skills to handle such situations. Propofol lowers intracranial pressure and can cause hypotension without an increase in heart rate. Green and Krauss evaluated its use in the pediatric ED and found satisfaction to be high among patients and parents. Due to the concern over inducing a state of deeper sedation than intended, they recommend using capnography and continuous oxygen during its use.[39] Havel *et al.* compared propofol to midazolam for sedation in the pediatric ED. They found that complication rates were comparable, but propofol had a clear advantage of having shorter recovery times.[29] Pershad and Godambe administered 1 mg/kg over 1-2 minutes or until eye closure and repeated 0.5 mg/kg over 30-60 minutes, recommending the use of syringe pumps for longer procedures.[41] Propofol is well-known to be painful upon injection, though the actual mechanism remains to be fully understood. It is postulated that the phenol group provokes the pain,[42] while others state it is the solvent that induces the kallikrein system and produces bradykinin.[43] To reduce the pain of propofol injection, 10 mg of lidocaine can be added to 100 mg of propofol. However, near-complete pain prevention has been attained with the use of a 50 mmHg tourniquet for one minute and administration of 2% lidocaine proposed by Mangar and Holak.[44] Propofol is titrated to maintain sedation and recovery usually occurs in 8 minutes, when patients are awake. Special care must be taken with the vials of propofol. No antibacterial agents are added to the propofol solution, so contamination and bacterial growth can occur relatively easily in the vial. Vials should be only used for a single patient. As noted above, the use of propofol may meet some resistance, despite its many positive attributes. Propofol can profoundly depress ventilation and even cause transient apnea following rapid infusion. Close patient monitoring and physician education is essential.[39,40]

### Nitrous oxide

Nitrous oxide (N_2_O) is a colorless, sweet-smelling, non-flammable gas that has both analgesic and sedative properties. At a concentration of 50%, nitrous oxide as the analgesic effectiveness of 10 to 20 mg of morphine. The gas is self-administered by the patient so that when the patient loses consciousness, the mask falls off. When used in children, an assistant or parent may help the child with the mask, but should not hold the mask continuously on the child's face. Rather they should watch the child's reaction and response to the gas to assess the level of sedation, only using the mask intermittently. The dose used for PSA is a mixture of 30-50% of nitrous oxide and oxygen. The onset of action is usually in two minutes and lasts about 5-10 minutes. Adverse reactions with nitrous oxide are minimal, but diffusion hypoxia can be serious. Normally only seen with nitrous oxide concentrations above 50%, diffusion hypoxia occurs as a result of the dissolved gas in the blood exiting through the alveoli, displacing the oxygen after rapid discontinuation of the oxygen. Nitrous oxide is contraindicated in patients that have a pneumothorax, eye injury, obstructed viscous, or an altered level of consciousness.[19,20,45]

### Combination formulas

Fentanyl/midazolam, morphine/midazolam, and ketamine/midazolam are three common combinations used in “balanced” PSA. When using an opioid and benzodiazepine, the opioid should be titrated to effect first followed by the benzodiazepine for further sedation. Kennedy *et al.* showed in one study that ketamine and midazolam was more effective in pain and anxiety relief and had less respiratory depression when compared to fentanyl and midazolam. However, the ketamine and midazolam combination group had a higher incidence of vomiting and recovery from sedation was longer.[46] The combination "DPT" or Demerol^®^ (meperidine), Phenergan^®^ (promethazine), and Thorazine^®^ (chlorpromazine) is no longer recommended, due to a high frequency of side effects, and the onset and depth of sedation that was very unpredictable.[28,46–48]

### Reversal agents

Naloxone is an antagonist of opioids at mu receptors. It can reverse the unwanted respiratory depression induced by opioids, like morphine and fentanyl. It does not, however, reverse the rigid chest wall phenomenon associated with fentanyl. If given to opioid-dependant patients, naloxone may induce withdrawal symptoms or pain. Flumazenil is a benzodiazepine antagonist and can safely reverse the sedative and respiratory effects caused by benzodiazepines. Even though flumazenil has been shown to bring patients safely out of midazolam-induced sedation, it is not recommended for routine use. The half-life of both naloxone and flumazenil is shorter than that of opioids and benzodiazepines and therefore require repeated administration of the antagonist until the sedatives have worn off.[13,20]

## SEDATION TEAMS

One of the new trends in ED sedation is to have a “procedural sedation” team. This notion is akin to “IV teams” already used in many hospitals. These teams consist of anesthesiologists and nurses which take care of all sedation outside of the OR. Gozal *et al.* evaluated one such team and found that it had high patient and staff satisfaction. However, they noted that it may not be feasible due to increased cost and limited availability. Though programs like these may serve large urban centers well, it is impractical for smaller institutions to house a devoted team of staff to only perform procedural sedation.[49]

## CONCLUSION

Procedural sedation and analgesia is one of the fundamental tools of the pediatric emergency department. It allows for good patient care and improves overall patient, parent, and staff satisfaction. There is some cost and training involved with safe and efficacious sedation, but the rewards are worth the efforts. PSA is a natural fit for the pediatric emergency department and should be utilized whenever clinically appropriate. Years of practice and multiple large studies have shown that when used properly, drugs used for PSA have low rates of complications. Though the use of propofol has met with some resistance, it appears to be an emerging drug in pediatric emergency departments as physicians become more familiar and comfortable with its use. Nevertheless, other medications, such as ketamine, fentanyl, etomidate, and midazolam, have proven their utility and safety over time.
